# Software-Defined Workflows for Distributed Interoperable Closed-Loop Neuromodulation Control Systems

**DOI:** 10.1109/access.2021.3113892

**Published:** 2021-09-20

**Authors:** PRADEEBAN KATHIRAVELU, PARISA SARIKHANI, PING GU, BABAK MAHMOUDI

**Affiliations:** 1 Department of Biomedical Informatics, Emory University, Atlanta, GA 30322, USA; 2 Department of Biomedical Engineering, Georgia Institute of Technology, Atlanta, GA 30332, USA

**Keywords:** Closed-loop simulations, neuromodulation control systems, workflow orchestration

## Abstract

Closed-loop neuromodulation control systems facilitate regulating abnormal physiological processes by recording neurophysiological activities and modifying those activities through feedback loops. Designing such systems requires interoperable service composition, consisting of cycles. Workflow frameworks enable standard modular architectures, offering reproducible automated pipelines. However, those frameworks limit their support to executions represented by directed acyclic graphs (DAGs). DAGs need a pre-defined start and end execution step with no cycles, thus preventing the researchers from using the standard workflow languages as-is for closed-loop workflows and pipelines. In this paper, we present *NEXUS*, a workflow orchestration framework for distributed analytics systems. *NEXUS* proposes a Software-Defined Workflows approach, inspired by Software-Defined Networking (SDN), which separates the data flows across the service instances from the control flows. *NEXUS* enables creating interoperable workflows with closed loops by defining the workflows in a logically centralized approach, from microservices representing each execution step. The centralized *NEXUS* orchestrator facilitates dynamically composing and managing scientific workflows from the services and existing workflows, with minimal restrictions. *NEXUS* represents complex workflows as directed hypergraphs (DHGs) rather than DAGs. We illustrate a seamless execution of neuromodulation control systems by supporting loops in a workflow as the use case of *NEXUS*. Our evaluations highlight the feasibility, flexibility, performance, and scalability of *NEXUS* in modeling and executing closed-loop workflows.

## I. INTRODUCTION

Designing, prototyping, and experimenting with neuromodulation control systems require implementing closed-loop analytic pipelines using interoperable modules. These systems can be modeled as several interacting *services* in computational environments [1]. Workflow languages and frameworks such as Common Workflow Language (CWL) [2] and Workflow Description Language (WDL) [3] revolutionize how eScience services interact with each other [4]. However, workflows are traditionally defined as a set of processes with a pre-defined start and a definite end service [5]. On the other hand, the control system workflows consist of feedback loops and are often without an explicit start and end step. Such workflows with closed-loops can be represented by directed graphs (DGs) that consist of directed cycles (dicycles). However, DG support from classic workflow frameworks and languages is marginal, if at all existent. This state of affairs prevents standard open-source workflow frameworks from modeling and implementing closed-loop neuromodulation control systems. Hence, we highlight how the representation of directed acyclic graphs (DAGs) in workflow languages commonly used in eScience is restrictive for various emerging research works on designing intelligent closed-loop neuromodulation systems that could leverage such automation and interoperability facilitated by a workflow framework.

### A. WORKFLOW ORCHESTRATION

With the proliferating number of workflow languages, researchers develop many scientific applications in various workflow languages. Enabling communication and coordination across existing workflows to compose a complex workflow is currently not a trivial undertaking due to incompatibility across workflow languages and the lack of an orchestrator that spans multiple scientific workflow frameworks. Furthermore, a workflow composed of several workflows can be more flexibly represented by a directed hypergraph (DHG) than a DG or a DAG [6]. However, such a dynamic representation is hindered by workflow definitions that tightly couple how the data flows between the services and the control of the services that compose the workflow.

### B. HYBRID CLOUD ENVIRONMENTS

Hybrid cloud infrastructures consisting of workflows seamlessly running on both cloud and local clusters have become more prevalent in recent days. The increasing reach of cloud computing has prompted many researchers to deploy their services in cloud environments to access them remotely and compose workflows from them. Major cloud providers such as Amazon Web Services (AWS), Google Cloud Platform (GCP), and Microsoft Azure provide their infrastructure and a platform to deploy and expose services (as well as the lightweight services known as microservices) quickly. Serverless cloud computing services such as AWS Lambda, Google Cloud Functions, and Apache OpenWhisk manage the resources for the end-users, thus letting the users focus entirely on the service deployment and management, rather than also having to configure the platform and infrastructure [7]. Cloud providers offer commercial hybrid cloud deployments such as Google Anthos [8] following the same on-demand payment policy of the cloud. Despite these advancements in the industry, scientific workflows are still developed in a single workflow language and deployed in a single infrastructure due to the cost, complexity, interoperability challenges, and potential vendor lock-in of such commercial offerings. Composing a complex workflow comprising smaller workflows developed in diverse frameworks deployed across various cloud infrastructures and research clusters remains a challenging undertaking. This problem is more prominent in the context of closed-loop neuromodulation systems, where there is a need for interfacing scalable neural data processing workflows in the cloud with local experimental and clinical settings.

### C. CONTAINERIZATION

Containerization technologies such as Docker and Singularity are used in developing the services to be portable, lightweight, and modular [9]. Containerization minimizes manual configuration efforts necessary to replicate a scientific research experiment [10]. Thus, containerized services are used in composing interoperable workflows to facilitate reproducible scientific research. Orchestration frameworks such as Kubernetes help researchers seamlessly compose and manage workflows from containerized microservices deployed across various infrastructures and platforms [11].

### D. MOTIVATION

Given the above premises and state-of-the-art research on workflow orchestration, hybrid cloud environments, and containerization, we aim at addressing the following research questions in this paper:

(*RQ*_1_) Can we formulate a flexible and dynamic workflow composition rather than building statically defined tightly-coupled workflows?
(*RQ*_2_) Can we compose workflows of more diverse definitions from existing services as well as workflows developed in various workflow languages and stand-alone microservices and represent them by a DHG?
(*RQ*_3_) Can we compose workflows from services and workflows implemented in various frameworks and deployed across multiple cloud and local infrastructures?
(*RQ*_4_) Can we develop and efficiently run closed-loop executions for various scientific use cases, such as neuromodulation control systems from existing physiological model services and workflows?

### E. CONTRIBUTIONS

This paper aims to answer the identified research questions. The main contributions of this paper are:

(*C*_1_) A Software-Defined Workflows approach, inspired by Software-Defined Networking (SDN), which separates the workflows’ control flows from the data flows to enable a dynamic workflow composition (*RQ*_1_).
(*C*_2_) A modular distributed approach to workflow composition that decouples complex workflows as workflows of workflows and allows representing complex workflows with dicycles as DHGs (*RQ*_1_ and *RQ*_2_).
(*C*_3_) A scalable and interoperable workflow orchestration framework that allows composing complex workflows from diverse workflow languages, service instances developed in various programming languages, and web service engines (*RQ*_3_).
(*C*_4_) A use case implementation of the workflow orchestration for closed-loop neuromodulation control systems (*RQ*_4_).

This paper proposes *NEXUS*, a flexible workflow orchestration framework that supports distributed analytics systems. We elaborate on modeling neuromodulation control systems as a use case of *NEXUS*. *NEXUS* incorporates template generation to convert a service that runs once into a service that runs in a dicycle while taking inputs from a centralized workflow management service that we call the *Orchestrator*. *NEXUS* aims to be flexible in both infrastructure-specific and application-specific aspects. From the infrastructure perspective, *NEXUS* supports services running across multiple execution environments such as Docker, Singularity, and directly on the operating system – locally as well as on a cloud platform.

### F. PAPER ORGANIZATION

The rest of the paper elaborates on *NEXUS* as a workflow orchestration framework for distributed closed-loop analytic pipelines. Section II presents the Software-Defined Workflows approach and the *NEXUS* workflow patterns. Section III presents the *NEXUS* architecture, algorithms, and prototype implementation. Section IV evaluates the *NEXUS* framework with a sample use case. Section V presents the state-of-the-art and related work. Finally, Section VI concludes the paper with a summary of the research and future work.

## II. THE *NEXUS* APPROACH

We design *NEXUS* as a workflow orchestration framework for closed-loop neuromodulation control workflows. This section introduces our novel Software-Defined Workflows approach and how *NEXUS* uses it to compose dynamic workflows.

### A. SOFTWARE-DEFINED WORKFLOWS

*NEXUS* consists of a modular architecture to natively support using services from multiple frameworks to compose workflows. It uses standard REST interfaces for its communications across service instances and between the service instances and the orchestrator. Leveraging standard REST interfaces and separating the data flow and control flow, *NEXUS* enables distributed workflows of various complexity beyond typical DAG workflows. Such a RESTful extension to scientific workflow frameworks also facilitates communications across workflow frameworks while enabling loosely coupled workflow definitions, providing dynamic workflow definitions.

*NEXUS* supports all the workflow patterns, including workflows with no loop (DAGs) and DGs consisting of simple or nested loops. *NEXUS* does not aim to replace any workflow languages or specifications. Instead, it seeks to fill a crucial void on developing closed-loop workflows in a distributed environment. *NEXUS* orchestrator can compose DHG workflows from scratch or exploit existing workflow definitions in standard workflow languages. Since CWL and WDL are commonly used workflow languages, *NEXUS* uses them as its primary workflow languages. It leverages complete and reference implementations of CWL and WDL such as Toil [12], CWL-Airflow [13], and Cromwell [14] to make DAG executions from the orchestrator. By default, *NEXUS* extends and exploits Cromwell [15] as its core workflow framework for WDL and CWL. We also tested *NEXUS* with CWL workflows executed with Toil, composed using Rabix front-end workflow composer. As standard WDL and CWL workflow frameworks offer a unified interface, *NEXUS* can coherently manage workflows with any of them without custom configurations. Therefore, *NEXUS* leverages a wide range of workflow frameworks with CWL and WDL support.

The *NEXUS* orchestrator lets the underlying workflow framework execute the DAG workflows. However, workflow frameworks cannot manage the DHG workflows independently by their design. The workflows with loops are represented by a DG or a DHG rather than a DAG. The orchestrator executes such workflows dynamically in a coordinated manner across multiple frameworks and infrastructures, thus supporting distributed workflows. By decoupling workflows as data and control flows, *NEXUS* facilitates building flexible distributed closed-loop analytic systems. We highlight such systems deviate from typical DAG workflows managed by standard workflow frameworks.

At the core of *NEXUS* is its Software-Defined Workflows approach that defines workflows dynamically, rather than following the static workflow definition approach of CWL or WDL (which do not change after the execution). This approach makes sense in typical DAG workflow scenarios, as those workflows have start and end steps and do not execute long enough to warrant a dynamic change. However, a complex workflow with a loop represented in a DHG can run longer as it traverses through its dicycles and runs the services in multiple iterations. During this time, a service instance may become less responsive, or the workflow definition could change dynamically. Any changes made to the workflow definition (typically specified in a file) must be dynamically represented in the workflow execution. A Software-Defined Workflows approach facilitates such a hot deployment of workflow definitions by managing and propagating the control of the workflows from the orchestrator.

**FIGURE 1.**
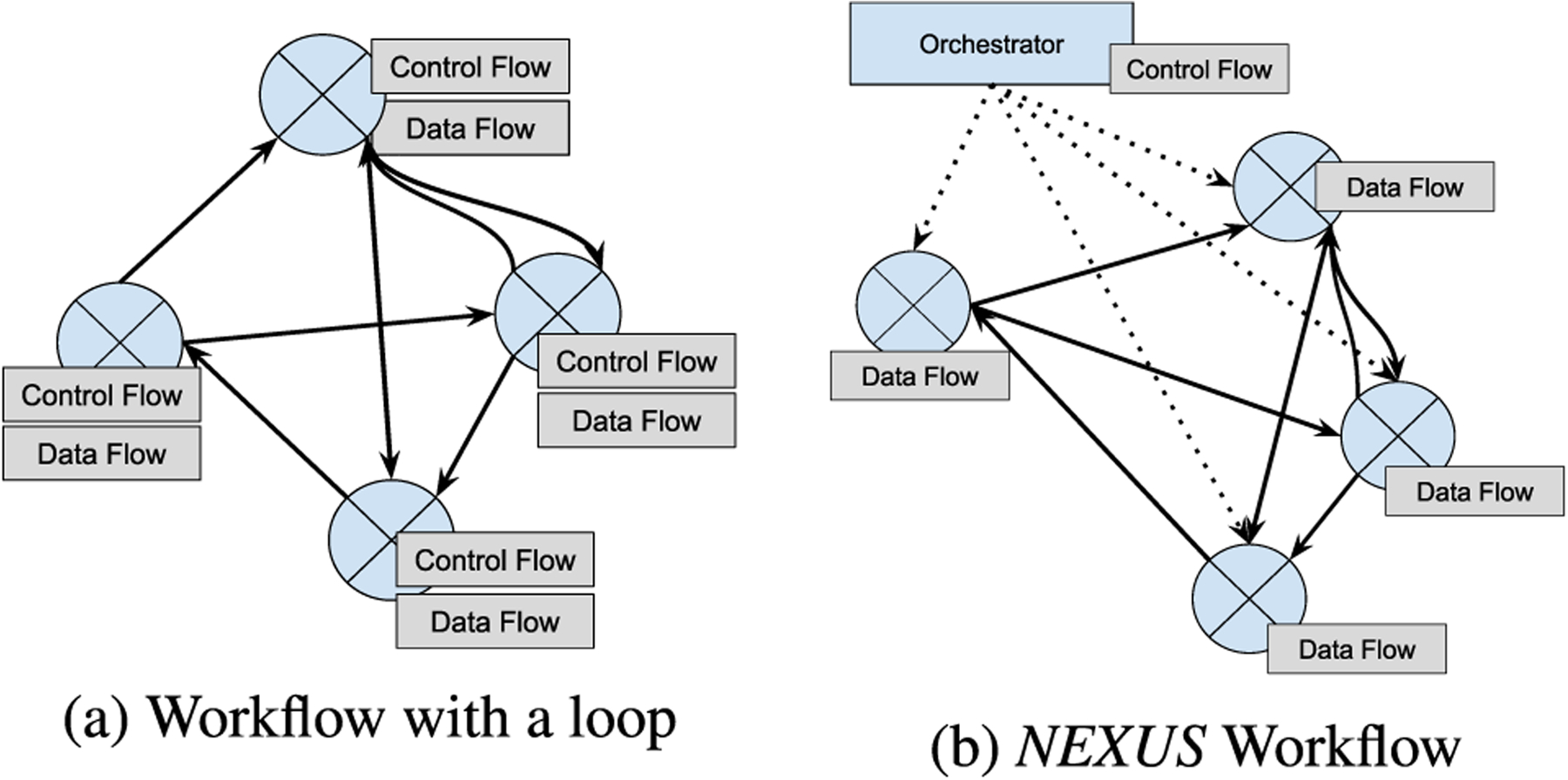
The separation of control flow from the data flow.

The Software-Defined Workflows approach separates the control and data flows as Figure 1 shows, to compose its workflows. The data flows with the large stream of actual data between the nodes in the data plane, abiding by the control logic mandated through the control plane. The control flows are between the nodes and the control plane through a northbound interface. The inspiration for such separation comes from SDN [16] where the control of the network switches is separated and unified into a logically centralized controller. SDN proposes a standard northbound interface such as OpenFlow [17] in the network switches to communicate with the controller. Leveraging the interface between the control plane and the data plane nodes, SDN and Software-Defined Workflows manage how the nodes send data between each other dynamically.

Such a separation of control enables dynamically modifying the workflows without changing the static definition of each service or workflow that is part of the complex workflow in runtime without downtime. *NEXUS* consists of a logically centralized service called the *orchestrator* that initiates the control flows that instruct how the data flow must happen. The orchestrator thus ensures services are chained according to the workflow specification.

### B. NEXUS NOTATION

*NEXUS* supports incorporating loops into existing DAG workflows or build DHG workflows from stand-alone services through its Software-Defined Workflow definitions. Table 1 summarizes the notation that we use in this paper to elaborate the *NEXUS* workflows.

**FIGURE 2.**
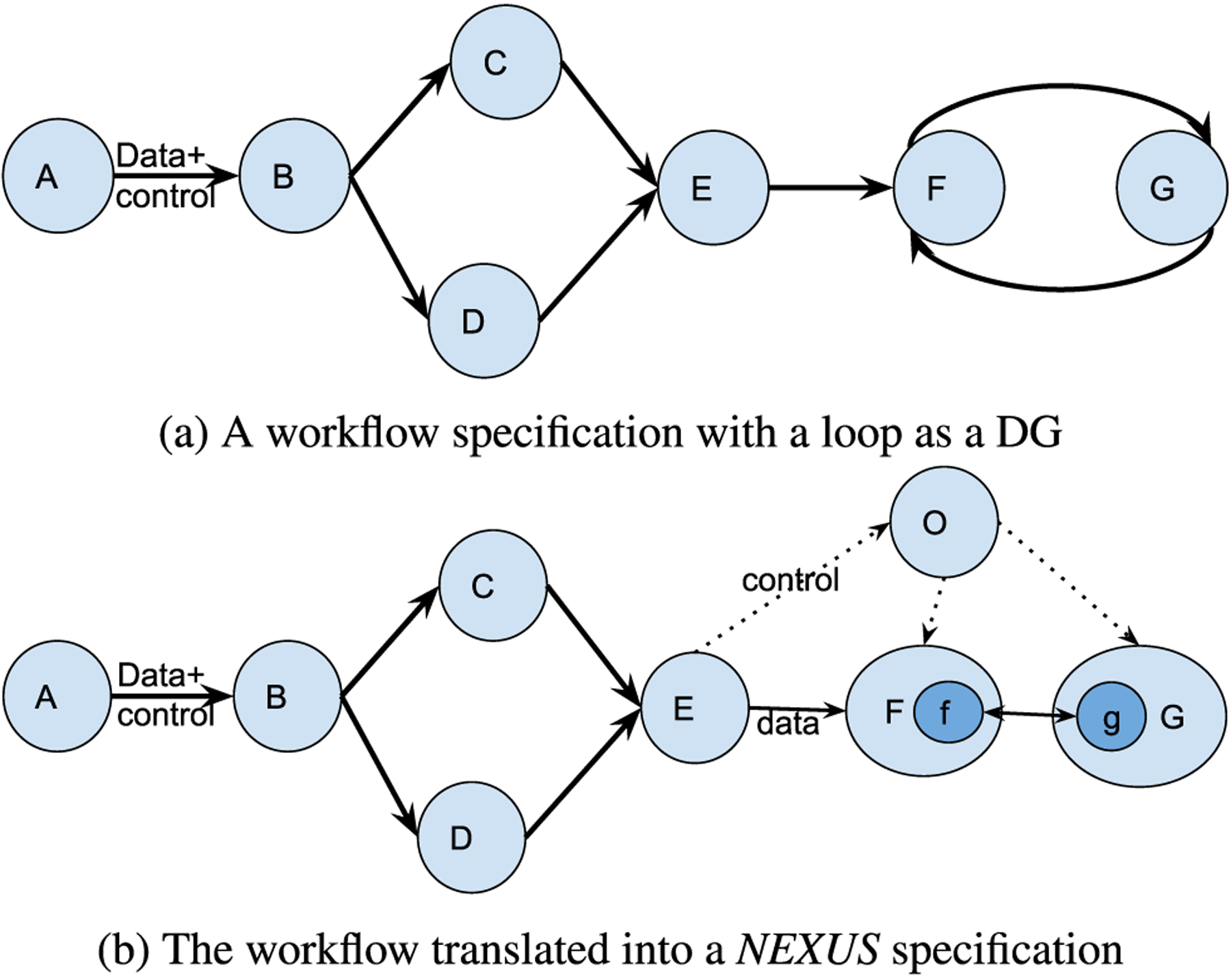
Converting the workflow with a loop into a *NEXUS* workflow.

Figure 2 illustrates a sample DG workflow (Figure 2a) and its *NEXUS* representation (Figure 2b). Here *NEXUS* modifies only a part of the workflow that includes the dicycle, leaving the first part of the workflow intact. Equation 1 denotes the Figure 2a workflow.

(1) W=A→B→(C+D)→E→F↔G

Figure 2b illustrates that the control flow in the transformed workflow is free from loops, whereas the data flow occurs orthogonally between the services. In the *NEXUS* transformation of DG workflows, the services without the loop and the segment of the DAG workflow are left unmodified.

A dicycle or a closed-loop breaks the tenet of a standard workflow that is expected to be represented by a DAG. The orchestrator as a node eliminates the loop from the control flow managed by the workflow manager, restoring the workflow representation in the control flow, thus enabling it to run in sequence regardless of the loop present in the original workflow representation. Nevertheless, unlike a DAG, a DG workflow with cycles may execute forever without an end step. As such, there should be an explicit exit condition to exit the loop, which we define to be met at the $N^{th}$ iteration. *NEXUS* describes this workflow with a closed-loop in its representation as Equation 2. The orchestrator starts the services F and G, defined as continuous “for loops” that take input from the other service (G and F) from their previous execution. Such a data transfer is handled orthogonally to the control flow managed by the workflow framework, thus not breaking the DAG workflow definitions.

(2) $$W=A\to B\to (C+D)\to E\to O\to (F+G)\;\exists N\in {\mathbb{Z}}^{+},\forall n\in {\mathbb{Z}}^{+},n\leq N\;f^{n}=F\left(G\left(f^{(n-1}\right)\right))\;g^{n}=G\left(F\left(g^{(n-1}\right)\right))$$

The services F and G run in a loop until a predefined exit condition is met or an update is sent from the orchestrator as a trigger event. The communication between the orchestrator and the services happens through a REST interface. The orchestrator functions as a RESTful web server while the service nodes act as lightweight REST clients. The changes and overhead to the service nodes are kept marginal through this loosely coupled lightweight implementation. The orchestrator uses its RESTful interface to send and receive updates as “events” (lightweight control messages) to indicate the completion of the iteration to start the next iteration and update the workflow.

Typical workflow frameworks such as Cromwell support running the workflow either locally or in a cloud environment. They do not let the users split the execution across multiple environments, such as a hybrid cluster, where a fraction of the workflow consisting of a few services runs locally, and the rest runs in the cloud. Similarly, they do not support multi-cloud workflow execution that spans multiple cloud and edge environments. *NEXUS* makes such an inter-infrastructure workflow possible through its decoupled workflows.

**FIGURE 3.**
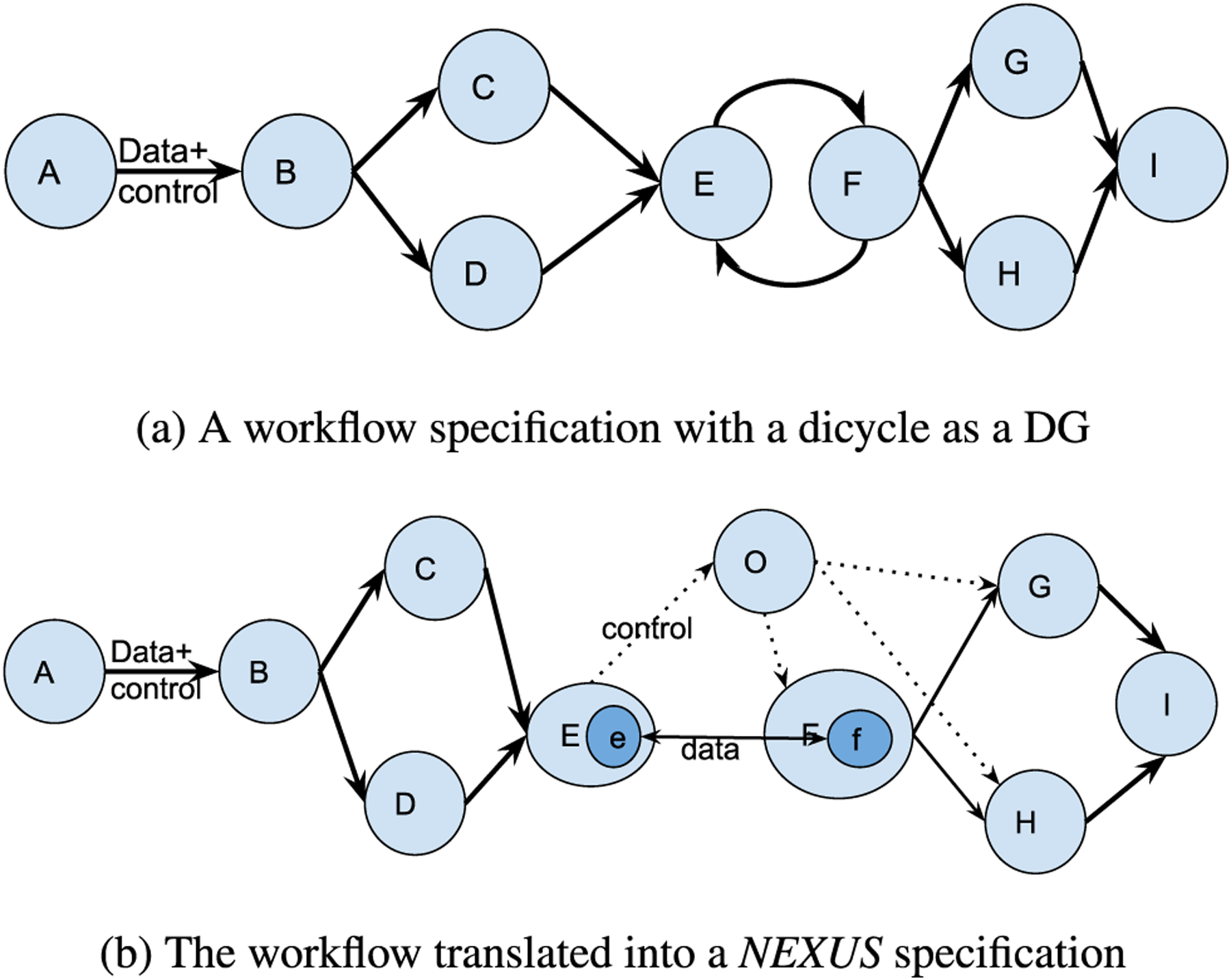
Converting a DG workflow with a dicycle into a *NEXUS* workflow.

Equation 3 depicts the workflow shown by Figure 3a. Adopting (2), *NEXUS* specifies this workflow as Figure 3b as illustrated by (4). This notion supports merging two or more workflows by chaining the first workflow’s outputs to another workflow as input parameters. As long as the final output(s) from the previous workflow can be chained to the input(s) of the following workflow, this design facilitates the inter-infrastructure composition of complex workflows from workflows running in different local and cloud environments.

(3) W=A→B→(C+D)→E↔F→(G+H)→I

(4) $$W=A\to B\to (C+D)\to E\to O\to (F+G+H),(G+H)\to I\;\exists N\in {\mathbb{Z}}^{+},\forall n\in {\mathbb{Z}}^{+},n\leq N\;e^{n}=E\left(F\left(e^{(n-1}\right)\right))\;f^{n}=F\left(E\left(f^{(n-1}\right)\right))$$

In *NEXUS* workflows that separate the data and control flows, the {data + control}, {control} flow segments define the workflow. The *NEXUS* workflow (4) can be depicted by two subworkflows *A* → *B* → (*C* + *D*) → *E* and (*G* + *H*) → *I* connected by the orchestrator to manage the loop in the middle. The subworkflows can run across multiple infrastructures, loosely connected via the orchestrator for control flows. The data flows between services that interact with the orchestrator utilize their RESTful interfaces in the Internet scale. If all the service nodes are local, they could leverage the memory or the file system to pipe the output of a service as an input to the next service. *NEXUS* leverages standard REST interfaces to the services and other similar mechanisms for data flows spanning various infrastructures.

### C. NEXUS WORKFLOW PATTERNS

**FIGURE 4.**
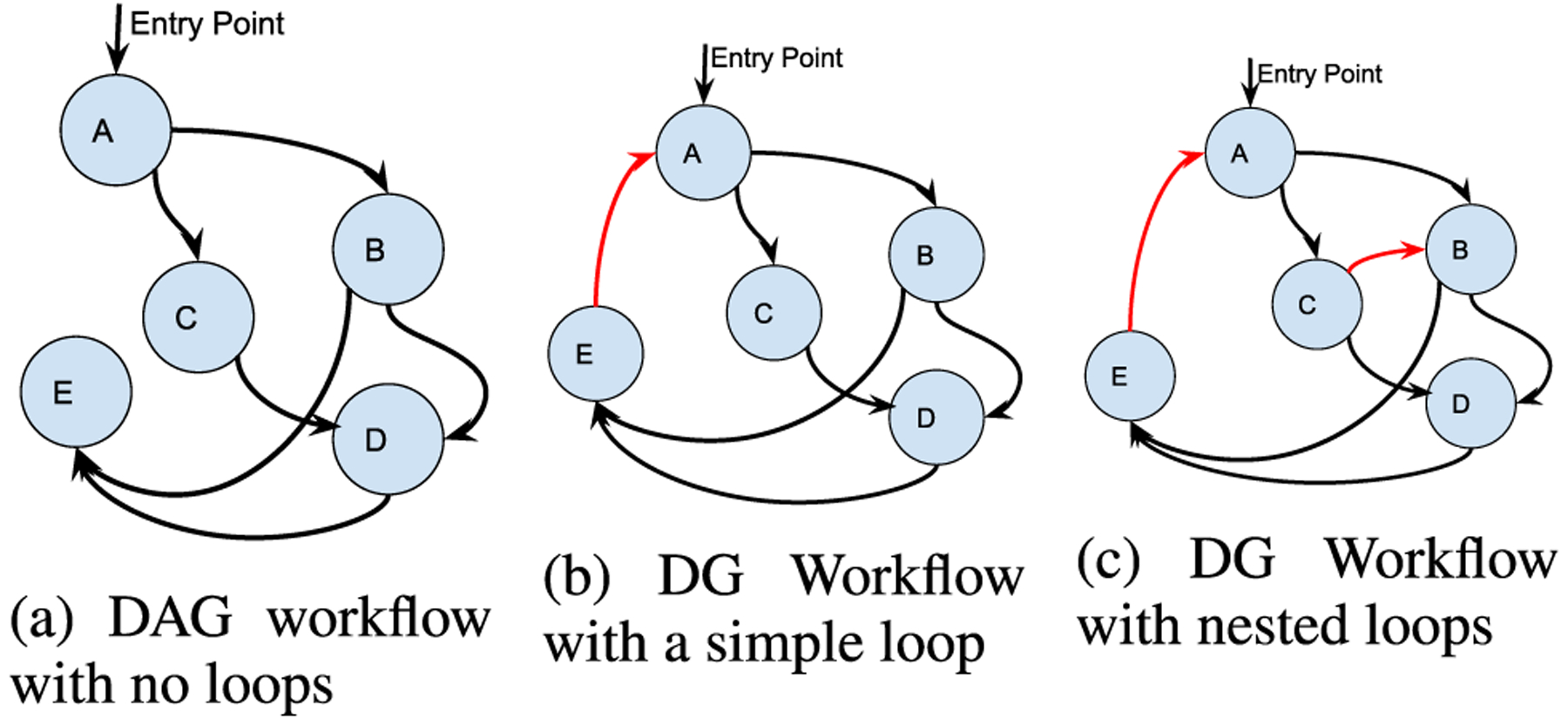
Synchronous variants of *NEXUS* workflows.

Figure 4 presents the synchronous workflow patterns of *NEXUS*. Here, each service waits for the output from its input services. Thus, every service executes once in each iteration step and exactly once in DAG workflows as in Figure 4a. Such a classic DAG workflow can be expressed in a standard workflow language such as CWL or WDL and run seamlessly in an existing workflow framework such as Toil [12] or Cromwell [14].

*NEXUS* “unrolls” a workflow, such as the one Figure 4b illustrates, as a *for loop* of a DAG that executes on a workflow framework such as Cromwell. The *NEXUS* orchestrator invokes the converted workflow in a *for loop*. Equation 5 refers to a simple loop with no split and merge.

(5) W=A→B→C→A→…

*ϕ*^*n*^ represents the *n*^*th*^ iteration of the service Φ, $\forall n\in {\mathbb{Z}}^{+}$, Φ ∈ {A, B, C, …}, *ϕ* ∈ {a, b, c, …}. Many workflow frameworks initialize a new service instance for each execution step and terminate upon completion. That means, in a loop, a service Φ will have several instances than the initial service instance of Φ. This approach of new service instances per execution makes the service node lose the context and local variables in a loop, as new instances of Φ, *ϕ*_*n*_ service instance for the *n*^*th*^ iteration of the workflow. In such a case, *ϕ*_*n*_ instances are instantiated across each iteration i of the loop. That means all the computed variables must be passed to the next nodes rather than storing any internally for the next iteration. Similarly, each service instance will consume time to initialize, thus adding up time in a DG. However, by pre-serving the same instance of a service instance across loops, the context of previous iterations can be saved, as shown by Equation 6. This approach minimizes the overhead of initialization time in *NEXUS*.

(6) $$\Phi =\phi_{n},\forall n\in {\mathbb{Z}}^{+}$$

We define *n*^*th*^ iteration of W as **w**^*n*^, $\forall n\in {\mathbb{Z}}^{+}$.

(7) $$w^{n}=C^{n}\circ B^{n}\circ A^{n}\left(w^{\left(n-1\right)}\right)$$

Equation 8 illustrates the same workflow as a series of functions, where i represents the initial input. When n = 1, **w**(*n* − 1) = **w**(0) = i.

(8) $$W=w^{N}\circ w^{\left(N-1\right)}\circ \ldots \circ w^{1}(i),\exists N\in {\mathbb{Z}}^{+}.$$

Equation 2 can be expanded to represent the workflow of Figure 4b.

(9) $$n=1\Rightarrow a^{1}=A(i)\;n>1\Rightarrow a^{n}=A\left(e^{(n-1}\right))\;b^{n}=B\left(a^{\left(n-1\right)}\right)\;c^{n}=C\left(a^{\left(n-1\right)}\right)\;d^{n}=D\left(c^{\left(n-1\right)},b^{\left(n-1\right)}\right)\;e^{n}=E\left(d^{\left(n-1\right)},b^{\left(n-1\right)}\right)$$

However, such a DG representation does not consider that the output can be split between the nodes. The output from A can all be sent to B and C or can be split between B and C if the output is composed of two separate outputs, for example, two output files, each respectively providing input to B and C. Therefore, a DHG representation optimizes the process by explicitly and natively indicating which outputs are identically sent. That is, if the output A → B = A → C, it can be represented by a directed hyperedge that connects [A, {B, C}], in a pair of {source, [destinations]}. A split output is represented by multiple edges rather than a hyperedge that connects a source to multiple destination service nodes.

Moreover, *NEXUS* limits its focus to hyperedges that can be represented by {source, [destinations]}, eliminating potential hyperedges that connect multiple services as in *A* → *B* → *C*, as they are natively represented by two different edges *A* → *B* and *B* → *C*. Thus, the *NEXUS* hyperedge definition mandates a lack of middle service between the source and destination in a hyperedge.

*NEXUS* supports DHG workflows using the most straight-forward means first. A workflow that a DAG can represent is always represented as such to enable portability between workflow frameworks and run the standard CWL and WDL workflows as-is. For workflows that cannot be defined by a DAG but only by a DG or a DHG, the workflow is deduced to the unrolling pattern (simple loop) or decoupling the workflows as control and data flows. The data flows orthogonally between the nodes when the data flows and control flows are decoupled. The orchestrator manages the control flow between the decoupled nodes that construct the loops and adjacent nodes. The orchestrator manages only a subset of nodes in a workflow that can be expressed as a combination of two or more DAG workflows. However, such an unrolling becomes infeasible for workflows with nested loops like the one presented in Figure 4c. The orchestrator coordinates such complex workflows entirely.

## III. SOLUTION ARCHITECTURE

**FIGURE 5.**
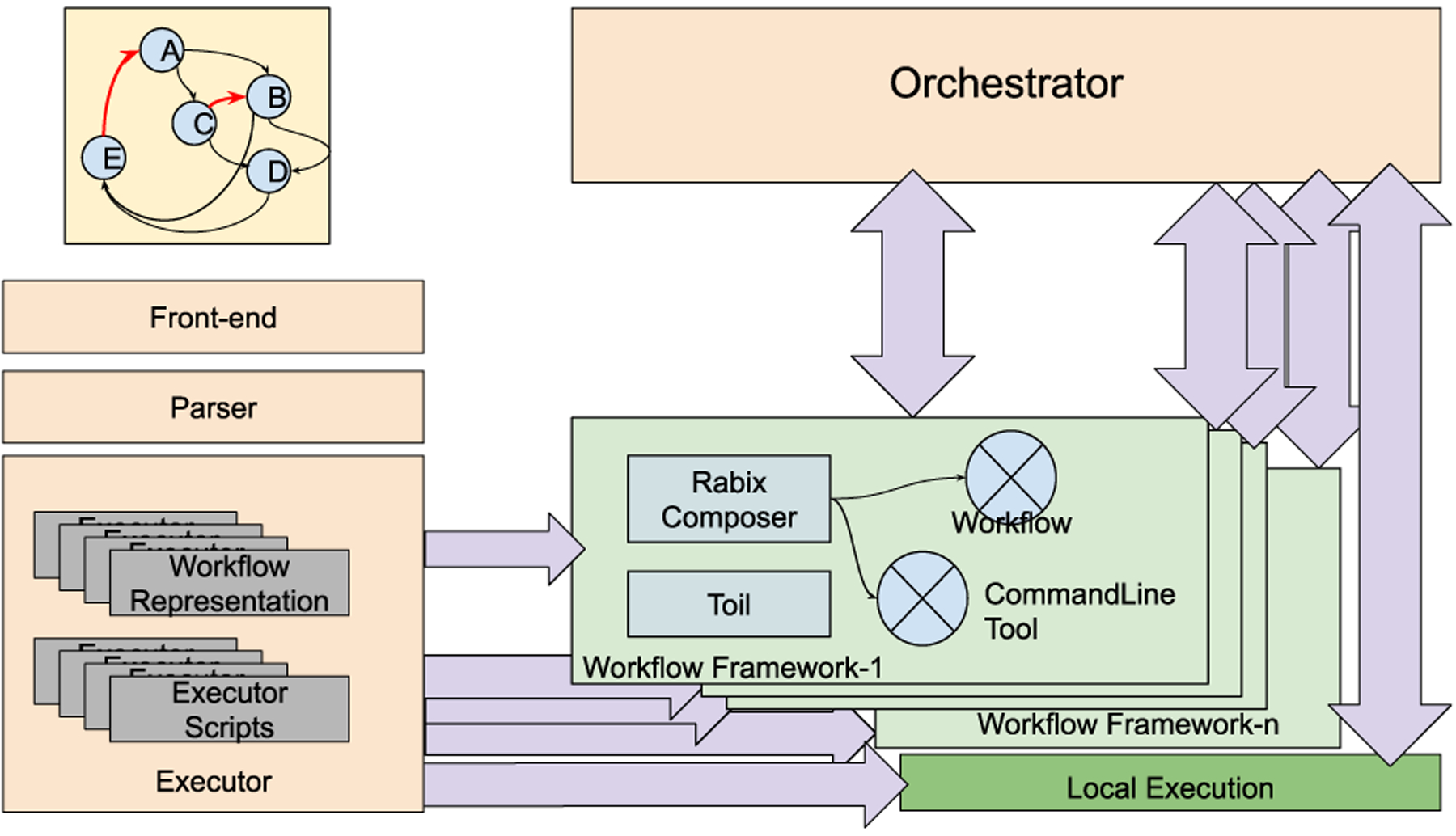
*NEXUS* deployment architecture.

Figure 5 depicts the architecture of *NEXUS*. In addition to the orchestrator and the workflow frameworks, *NEXUS* consists of a front-end, a parser, and an Executor. The front-end lets the users visually compose their workflows. The parser parses them into workflow representations and executor scripts for the Executor. The *NEXUS* Executor initiates the workflows, communicating with the *NEXUS* Orchestrator. The Orchestrator and Executor start first, followed by the services of the workflow, as represented by a DHG in the front-end. Since DAGs and DGs are subsets of DHGs (DAGs ⊂ DGs ⊂ DHGs), supporting DHG allows a more inclusive representation.

**FIGURE 6.**
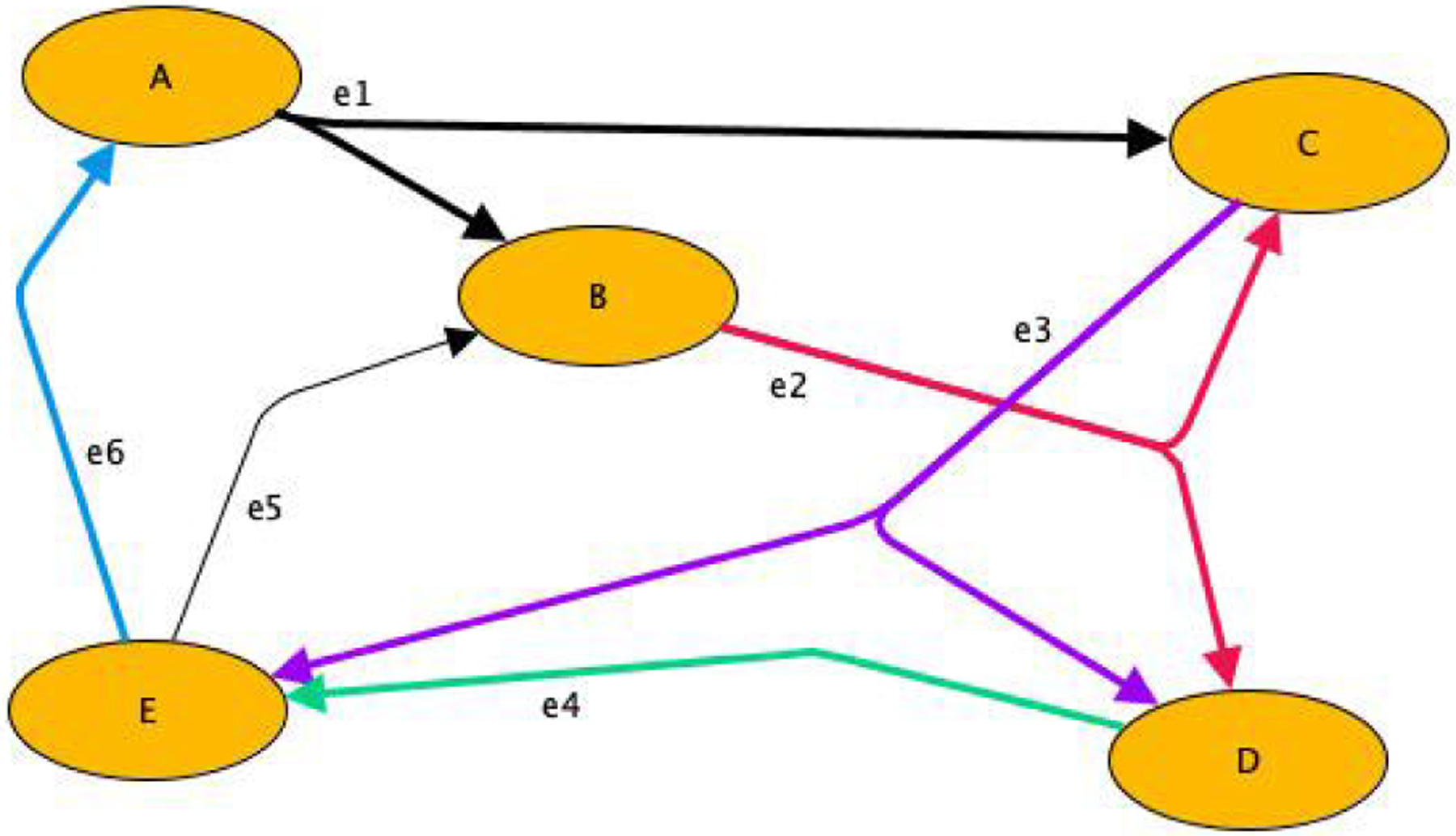
Visual development of a *NEXUS* workflow.

*NEXUS* utilizes its front-end to compose drag-and-drop DHG workflows with node and edge labels as Figure 6 illustrates. *NEXUS* imposes specific requirements to facilitate a complete workflow definition through its front-end. Each node and hyperedge is labeled in a *NEXUS* workflow. For example, an edge could connect a source to multiple destinations, indicating that the same output is sent from the source to various destinations.

We develop the workflows using the front-end, which stores the workflow definitions in an XML format. A simplified XML representation of Figure 6 is shown below:

<graph>
 <node id=“A” entrypoint=“True”>
  <edge id=“e1”>
   <node id=“B” />
   <node id=“C” />
  </edge>
 </node>
 <node id=“B”>
  <edge id=“e2”>
   <node id=“C” />
   <node id=“D” />
  </edge>
 </node>
 <node id=“C”>
  <edge id=“e3”>
   <node id=“D” />
   <node id=“E” />
  </edge>
 </node>
 <node id=“D”>
  <edge id=“e4”>
   <node id=“E” />
  </edge>
 </node>
 <node id=“E”>
  <edge id=“e5”>
   <node id=“B” nonblocking=“True”/>
  </edge>
  <edge id=“e6”>
   <node id=“A” />
  </edge>
 </node>
</graph>


In the representation, the thick edges indicate the blocking synchronous service executions, the default behavior in a workflow definition. A service execution waits for the new input from the previous service node before the current node executes, blocking the execution until then. The thin edges are non-blocking, representing asynchronous executions. The destination service nodes of such edges continue the execution without waiting for any input from the other service node instances. When an updated value is available to an asynchronous service node, it updates the local variables accordingly for the subsequent service execution.

The workflow starts with service *A*. *A* consists of the initial values for the first iteration, but the initial values are altered by the input from *E* for the subsequent iterations. Until it gets the input from *E*, *A* does not start the subsequent iteration. The hyperedge *e*1 sends the output of *A* to *B* and *C*. The service nodes *B* and *C* wait for the same output from *A* to initiate their current service execution. Then *e*2 sends the output of *B* to *C* and *D*. As earlier, *C* and *D* wait for the value from *e*2 for their execution. Similarly, *e*3 sends the output of *C* to *D* and *E*, and *e*4 sends the output of *D* to *E*. All the above service nodes wait for the output from the previous service nodes to start their current iteration. *e*5 sends the output of *E* to *B* in a non-blocking manner, asynchronously, unlike the rest of the edges. Therefore, while *e*5 alters the respective values defined in *B*, *B* does not wait for this input to continue its current iteration. *e*6 sends the output of *E* to *A* synchronously as in the case of all the edges in this sample DHG workflow except for *e*5. Data from *e*6 completes one iteration of the workflow, starting the next iteration with *A*.

The parser converts the XML representation (such as the one demonstrated above) into the representation of {source, [destinations]}. The workflow representation and executor scripts are then sent to the workflow engines or executed natively by the *NEXUS* Executor. The Executor contains utility functions to support data flow or to invoke the workflow frameworks. The orchestrator then manages the workflows as they are executed by the workflow frameworks or natively on the infrastructure. The orchestrator consists of a REST interface to communicate with the services. The services have a REST interface or other standard messaging/communication mechanism to share data among themselves. For the segments of the DHG workflows that are managed by the workflow composer frameworks such as Toil or Cromwell, such data flow is handled by those frameworks, respectively.

### A. THE NEXUS ORCHESTRATOR

As the orchestrator is a logically centralized entity, its performance is crucial for the scalability of *NEXUS*. Separation of control flows and data flows enables changing the paths from the orchestrator based on dynamically changing workflow definitions and other contextual variables. The orchestrator propagates these changes as *events*, light-weight control messages. The orchestrator tracks the workflows as control flows while letting the data flows between the service instances. Hence, the orchestrator can dynamically change the workflow through the control flows with its REST interface. The REST interface functions as the standard API for the management of workflows. *NEXUS* separates the orchestrator from an Executor that initializes the workflows. It thus facilitates interoperability and backward compatibility with the standard DAG workflow definitions by letting the executor perform the workflow executions entirely when the workflow definitions meet the DAG format currently supported by the standard workflow frameworks.

Algorithm 1 summarizes the execution of a *NEXUS* workflow from a user’s perspective. It starts with parsing the user’s visual definition of the workflow into the Workflow Representation (wfRepresentation) and Executor Scripts (execScripts) (lines 2 – 3). A unique workflow ID (wfID) is generated as a hash of the workflow representation (line 4). The *NEXUS* executor initializes with the values of wfID, wfRepresentation, and execScripts to start the workflow (line 5). Once the workflow is parsed into the executor, the executor converts the wfRepresentation into *NEXUS* workflow representation (*nexusWorkflow* in line 6), as Equation 4 shows.


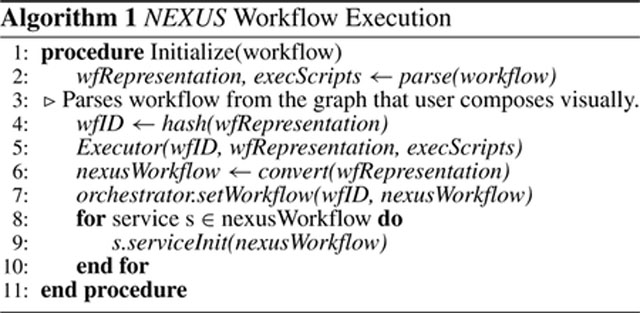


The nexusWorkflow remains the same as the workflow representation (wfRepresentation) for DAG workflows. The executor uses the workflow definition to transform the workflows with loops (i.e., the subset of DHG workflows that cannot be represented as DAGs) into *NEXUS* workflows involving the orchestrator. The executor starts the workflow execution based on these parameters and how the system is configured, including the default workflow frameworks to execute and the access to the execution infrastructures. The executor then sets the workflow on the orchestrator so that the orchestrator holds the initial definition of the workflow (line 7). Then the executor starts the workflow by initializing the services that compose the workflow (line 8 – 10). The workflow frameworks perform the execution directly for DAG workflows and the workflows with a DAG component as their startup nodes.

The workflow frameworks manage the typical DAG workflows without additional inputs from *NEXUS*. However, workflows with a closed-loop require inputs as events from the orchestrator to make such loops and a DHG workflow feasible. Algorithm 2 presents the execution of each service instance.


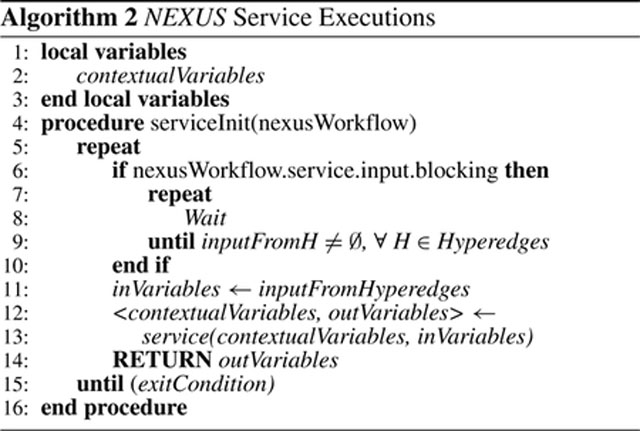


Every service node in a workflow consists of contextual variables stored locally and updated with each iteration of the service execution (lines 1 – 3). A hyperedge can denote synchronous and asynchronous executions. Synchronous execution represents where the execution waits until an updated value from a connecting hyperedge from a previous service node is received (lines 6 – 10). The service execution does not need to wait for such input in asynchronous executions, unlike synchronous executions. The input variables are from the hyperedges where the current service node is a destination (line 11). A service execution updates these variables (local contextual variables and output variables for the subsequent service nodes), considering the values received from the other service nodes and the local contextual variables (lines 12 – 13). The output is sent to the subsequent service nodes through the connecting directed hyperedges where the current node is the source (line 14). The service execution continues until an exit condition is met, as per the workflow definition and the current values of the variables or as an event from the orchestrator (line 15).

### B. REPRESENTATIVE USE CASE

**FIGURE 7.**
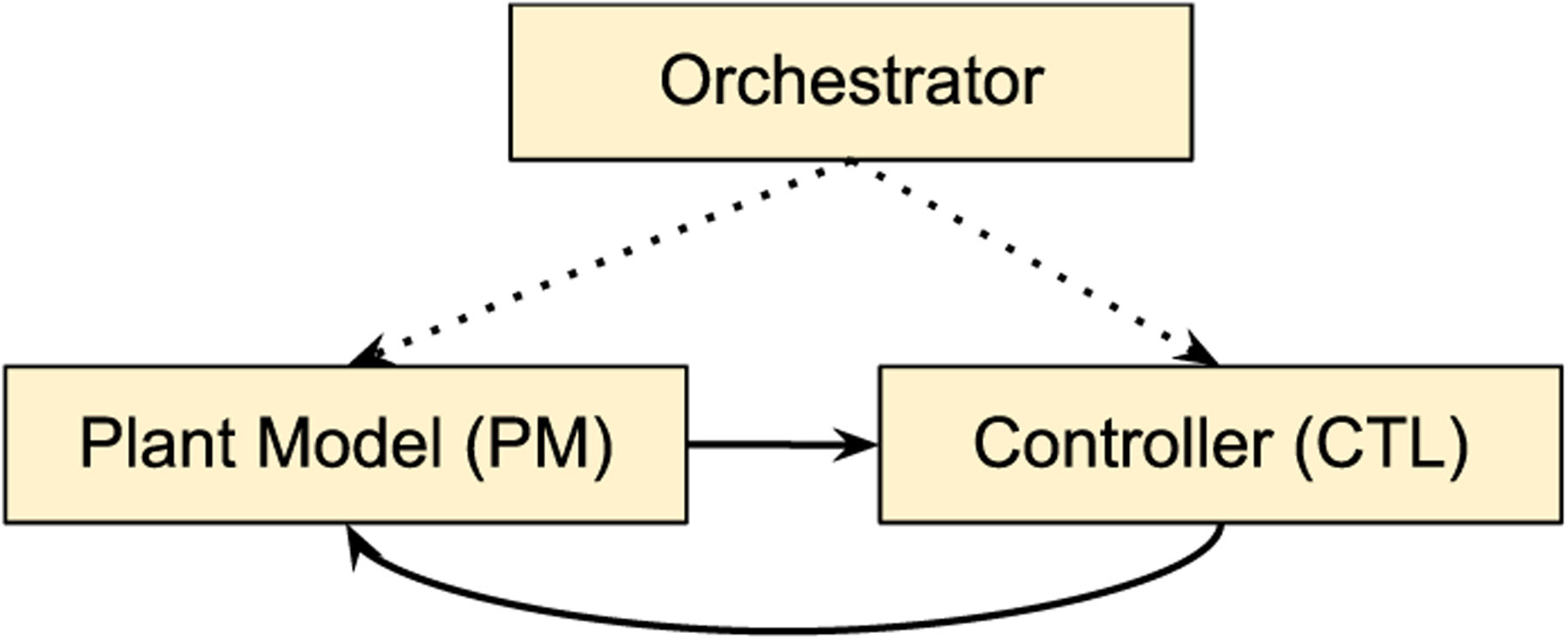
Simplistic sample use case of a *NEXUS* workflow.

As a *NEXUS* use case, we build a closed-loop neuromodulation control system workflow that aims to maximize the gamma-band power of the excitatory population, in a computational model of the brain, by applying electrical stimulation with proper parameters. A minimal workflow of a closed-loop neuromodulation control system contains two main modules. First, the plant model (PM) enables applying stimulation signals and observing the parameter-dependent effects of the interactions between the stimulus and the endogenous oscillations of the nervous system. Second, the controller (CTL) or optimizer, which is at the core of closed-loop neuromodulation control systems, tunes the stimulation settings based on measured objective values. We model this workflow entirely with *NEXUS* without workflow frameworks as it is just two nodes interacting via the orchestrator, as Figure 7 shows.

The plant model is a biophysically grounded mean-field model of neural populations under electrical stimulation [18] that can be used to efficiently study the effects of electrical stimulation on large neural populations. We leverage Bayesian optimization [19] in closed-loop with the mean-field model to optimize the parameters of stimulation, i.e., amplitude and frequency, to maximize the gamma-band power of the excitatory population. We implement Bayesian optimization using the GPflowOpt [20] library.

Bayesian optimization is a global optimization algorithm suitable for cases where the objective function is unknown or expensive to evaluate. Bayesian optimization finds the optima through a two-step and sequential decision-making process. First, it builds a surrogate Gaussian Process Regression (GPR) model using the collected data and then suggests the next candidate points to be evaluated by optimizing an underlying acquisition function. We used the common upper confidence bound acquisition function. During the burn-in phase, the controller module, i.e., Bayesian optimization, takes random actions over the parameter space, i.e., stimulation amplitude and frequency, by sending stimulation parameters to the plant model. The plant model returns the objective values, i.e., *γ*-band power of excitatory population, corresponding to each set of stimulation parameters to the controller. Next, Bayesian optimization builds a surrogate GPR model on the collected data. Then, it suggests the next set of parameters be evaluated on the plant model by optimizing the surrogate-dependent acquisition function. This sequential process of the interactions between the plant and controller modules continues until convergence or a predefined number of iterations.

*NEXUS* enables dynamically composing workflows without tightly coupling the service nodes such as PM and CTL to each other. *NEXUS* workflows are loosely defined and connected via the orchestrator. The loose coupling and dynamic formation of *NEXUS* workflows support seamless migration of workflows from local deployments to hybrid and multi-cloud environments. We evaluate this use case for the performance of *NEXUS* executed in hybrid clouds (one service node instance running locally with others in cloud instances) and multi-clouds (service nodes spanning multiple cloud provider instances).

## IV. EVALUATION

We evaluate the performance of *NEXUS* for its features and performance in a closed-loop neuromodulation task. In terms of the computation time, we assess *NEXUS* against a varying problem size and concurrency to understand how it scales.

### A. USE CASE PERFORMANCE

We configured the plant model (PM) and controller (CTL) in a closed-loop to communicate via an orchestrator. We deployed the CTL locally on an x86 laptop (2.8 GHz CPU, 16 GB memory, and macOS Big Sur operating system) in Atlanta, GA, USA. We deployed the PM on an HP Proliant DL320e Gen8 (E3-1240v2) 4-LFF server (3.4 GHz – turbo up to 3.8 GHz CPU, 16 GB memory, and Ubuntu 20.04 operating system) managed by Voxility infrastructure provider in Bucharest, Romania. Finally, we deployed the orchestrator on an AWS cloud instance (instance type t3.medium, 2 vCPU, 4 GB memory, and Ubuntu 20.04 operating system) in North Virginia to orchestrate the workflows that communicate their data updates through the orchestrator.

**FIGURE 8.**
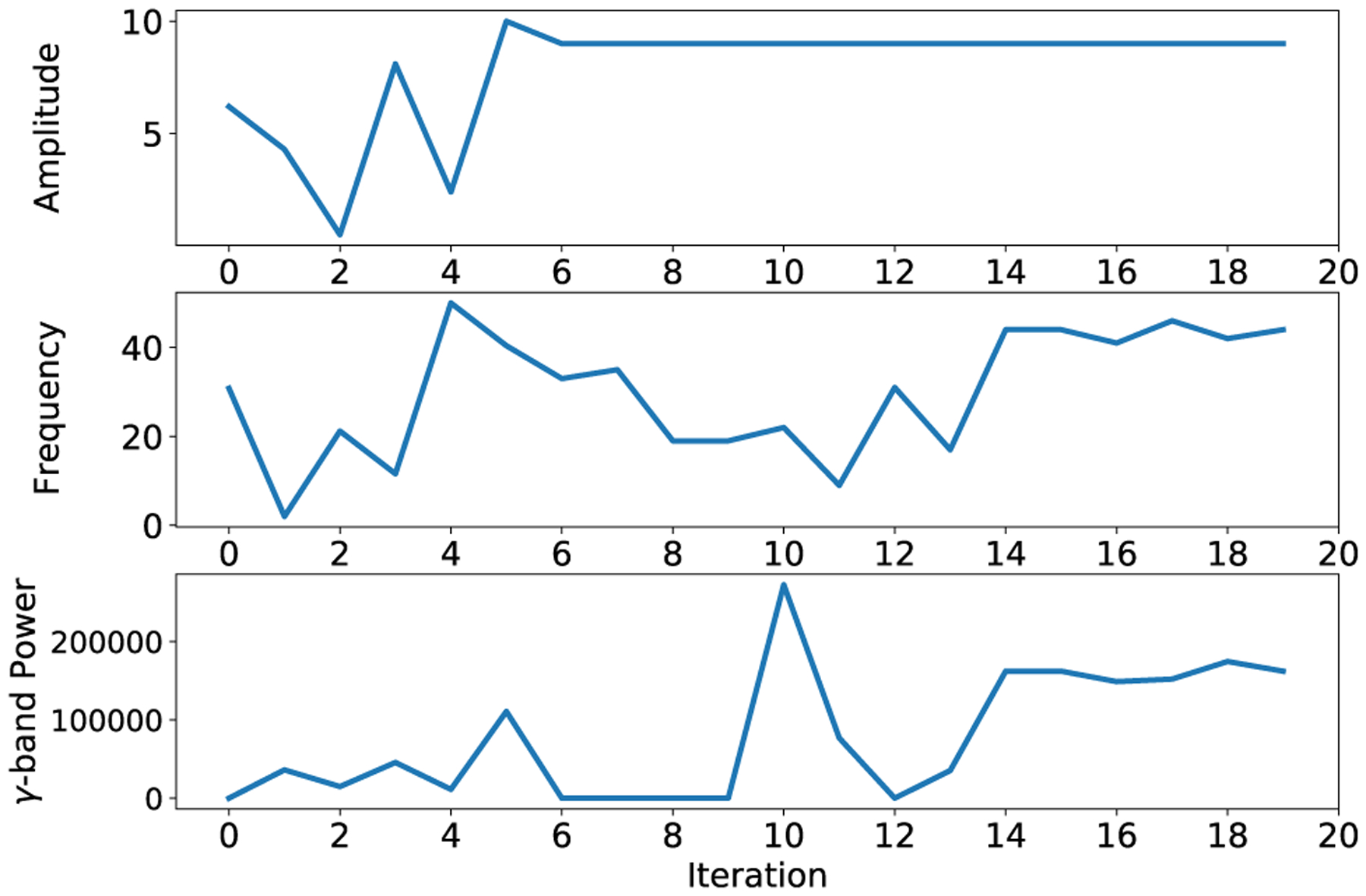
Performance of Bayesian optimization in searching for the stimulation parameters that maximizes the objective value, i.e. *γ*-band power over 20 iterations. The trajectory of parameters, i.e. stimulation amplitude and frequency, collected and their corresponding *γ*-band power is shows from top to bottom, respectively.

This evaluation aims to serve as a sample problem and demonstrate distributed directed graph workflows spanning a local server/laptop, a remote server from a cloud provider (AWS), and a distant remote server from an infrastructure provider (Voxility). Figure 8 illustrates the performance of Bayesian optimization in maximizing the objective, i.e., *γ*-band power over 20 iterations of interacting with the mean-field model of a neural population under electrical stimulation. The first five iterations are the burn-in phase, where the controller takes random actions over the parameter space and observes the objective values, i.e., *γ*-band power. After initialization, Bayesian optimization suggests the subsequent samples that should be evaluated. The trajectory of parameters, i.e., stimulation amplitude and frequency, collected and their corresponding *γ*-band power is depicted in Figure 8.

**FIGURE 9.**
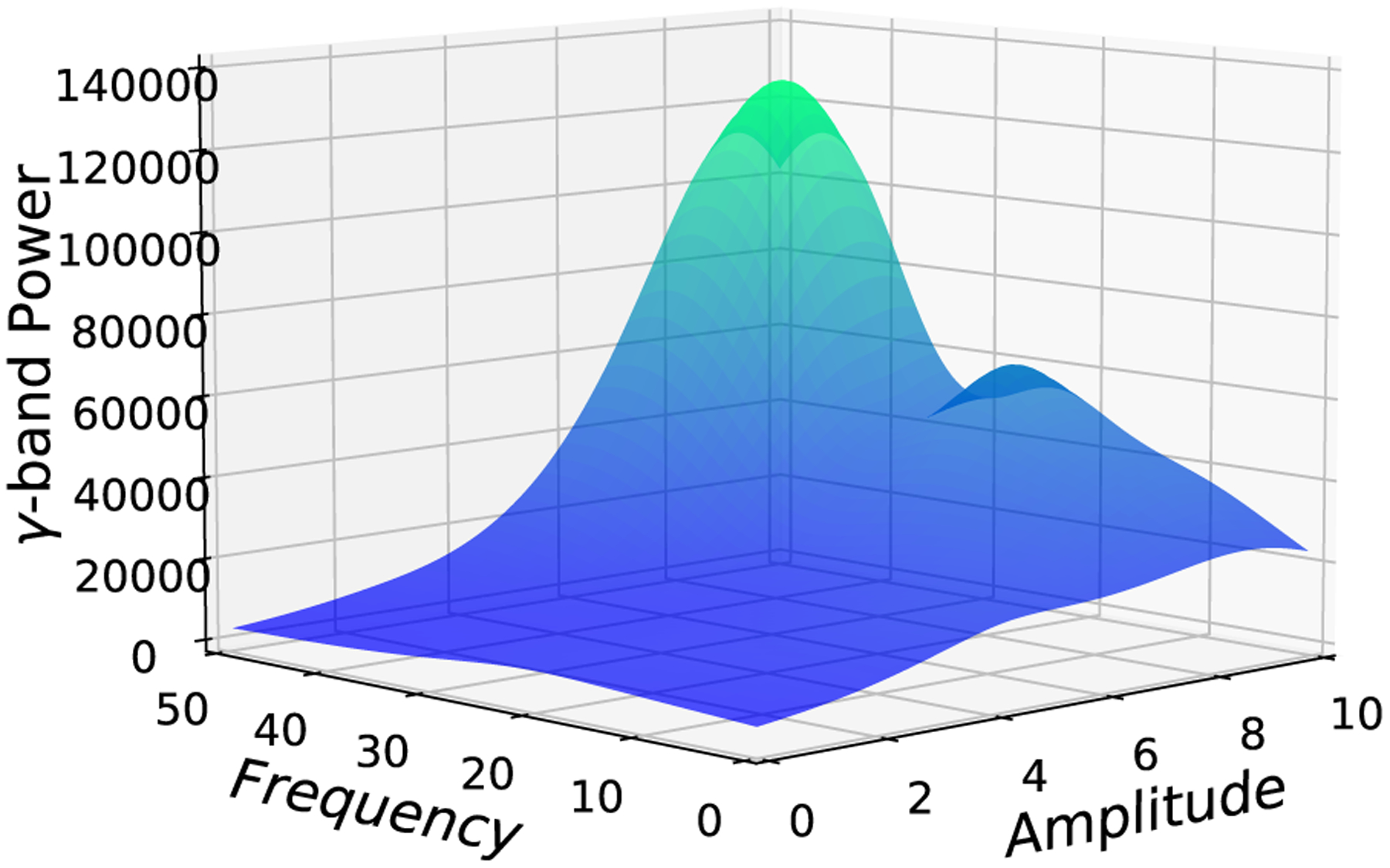
Mean surface of the surrogate GPR model, where the z-axis shows the objective, i.e. *γ*-band power over the parameter space. x- and y-axes show the frequency and amplitude of stimulation.

Figure 9 shows the mean surface of the surrogate GPR model, where the z-axis shows the objective, i.e., *γ*-band power over the parameter space. x- and y-axes show the frequency and amplitude of stimulation which are the inputs of the plant model.

**FIGURE 10.**
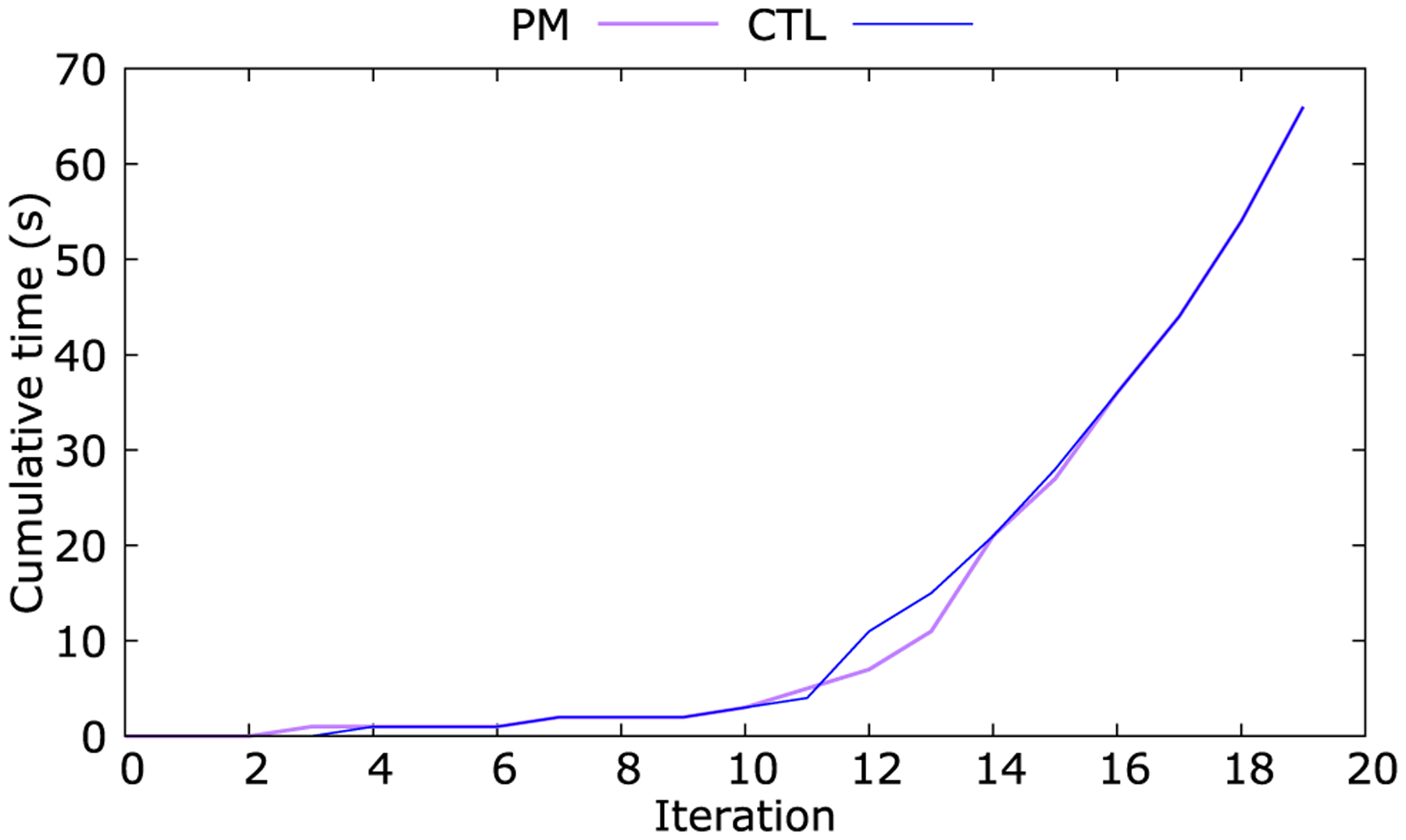
Cumulative execution time with iterations.

Figure 10 shows the cumulative execution time with iterations for both PM and CTL. We observe the initial time for the burn-in phase for each iteration to be much lower. After this initialization, we notice a linear execution time. No overhead is imposed by the *NEXUS* orchestrator in executing the closed-loop workflow between the PM and CTL in a distributed manner, compared to running the workflow as a centralized monolith in a single deployment infrastructure.

### B. CONCURRENCY AND SCALABILITY

We then benchmark the performance and scalability of *NEXUS* through a load test to emulate larger complex concurrent workflows. *NEXUS* exhibits high scalability in its data plane consisting of service nodes as they are distributed across several servers. The orchestrator processes all the control flows from the services distributed across several nodes. Thus, the orchestrator operates as a centralized entity. We can extend the orchestrator to run in a distributed cluster while it remains logically centralized. However, that requires an additional development effort. Therefore, we evaluate a stand-alone deployment of the orchestrator for its capability to manage multiple workflows at once without incurring bottlenecks, overheads, and failures. We assess how many control flows the orchestrator can handle simultaneously by executing multiple service workflows at once. We monitor the performance of several concurrent workflows managed by the orchestrator on an AWS cloud VM, invoked over the Internet from the laptop and the Voxility server. We configure Apache JMeter [21] to evaluate the efficiency of *NEXUS* orchestrator to manage concurrent workflows. While replacing the workflow with multiple REST clients to the orchestrator, we use the same distributed cloud deployment to emulate several large workflows.

**FIGURE 11.**
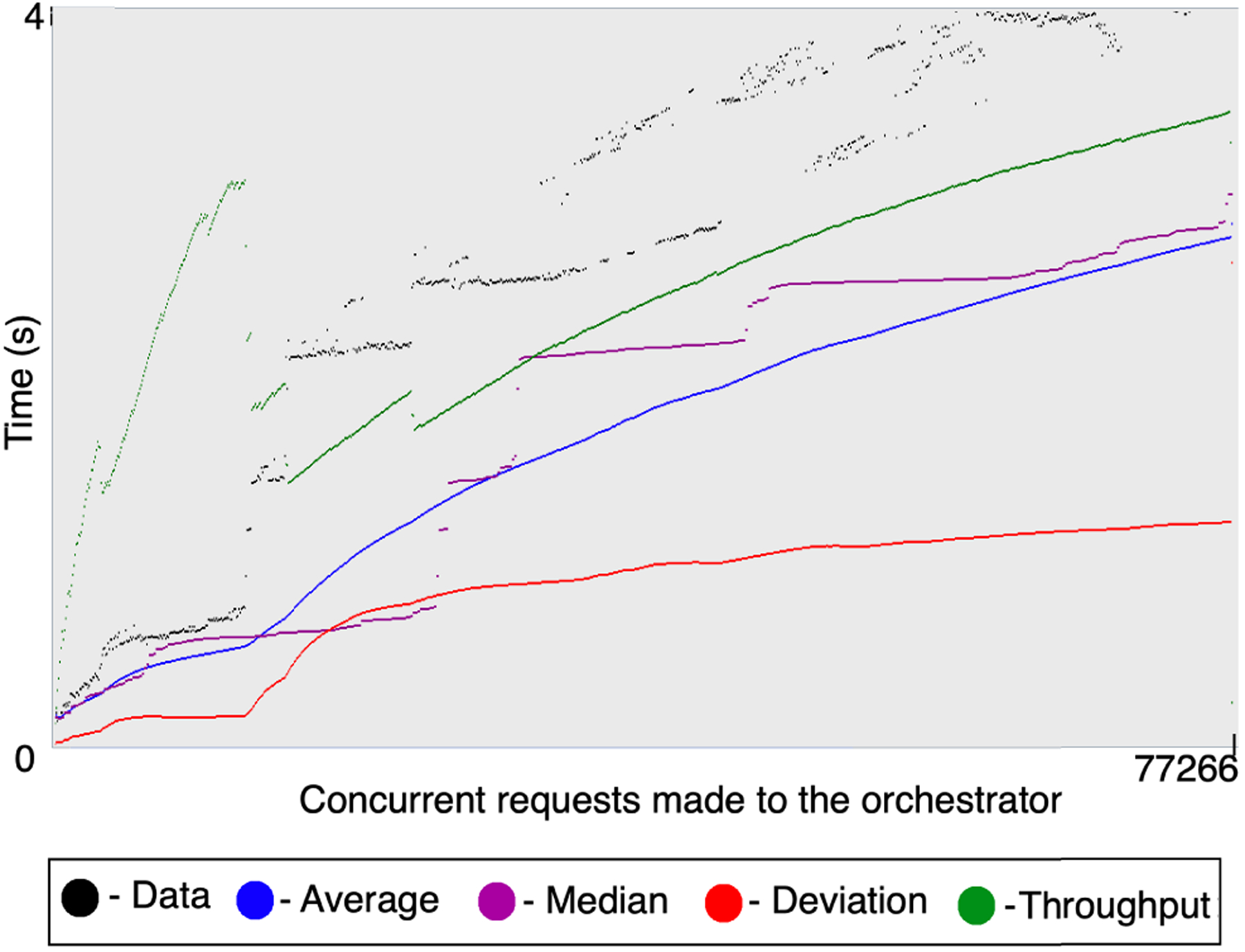
Execution of 1000 concurrent service workflows via the *NEXUS* orchestrator.

We observe that the orchestrator manages the workflows with several concurrent requests efficiently. Figure 11 shows the performance with 1000 such service workflows managed by the orchestrator through its REST interface. With 1000 concurrent invocations with a startup period of 1 s for all the invocations, we observe the throughput to be 768.512 workflow invocations per minute. On average, the service invocation from the orchestrator takes as low as 2.868 s and up to 4.037 s. The median time is 3.030 s with a deviation of 2.654 s. The actual workflow execution time will vary and take longer based on the time to invoke and complete each service in the workflow. The overhead due to the Internet-based *NEXUS* orchestrator is a fraction of a second. Thus, the orchestrator supports distributed execution of modular workflows with no added overhead in its dynamically defined concurrent workflows. *NEXUS* managed to execute all the 1000 workflows successfully, with no data loss or failures.

**FIGURE 12.**
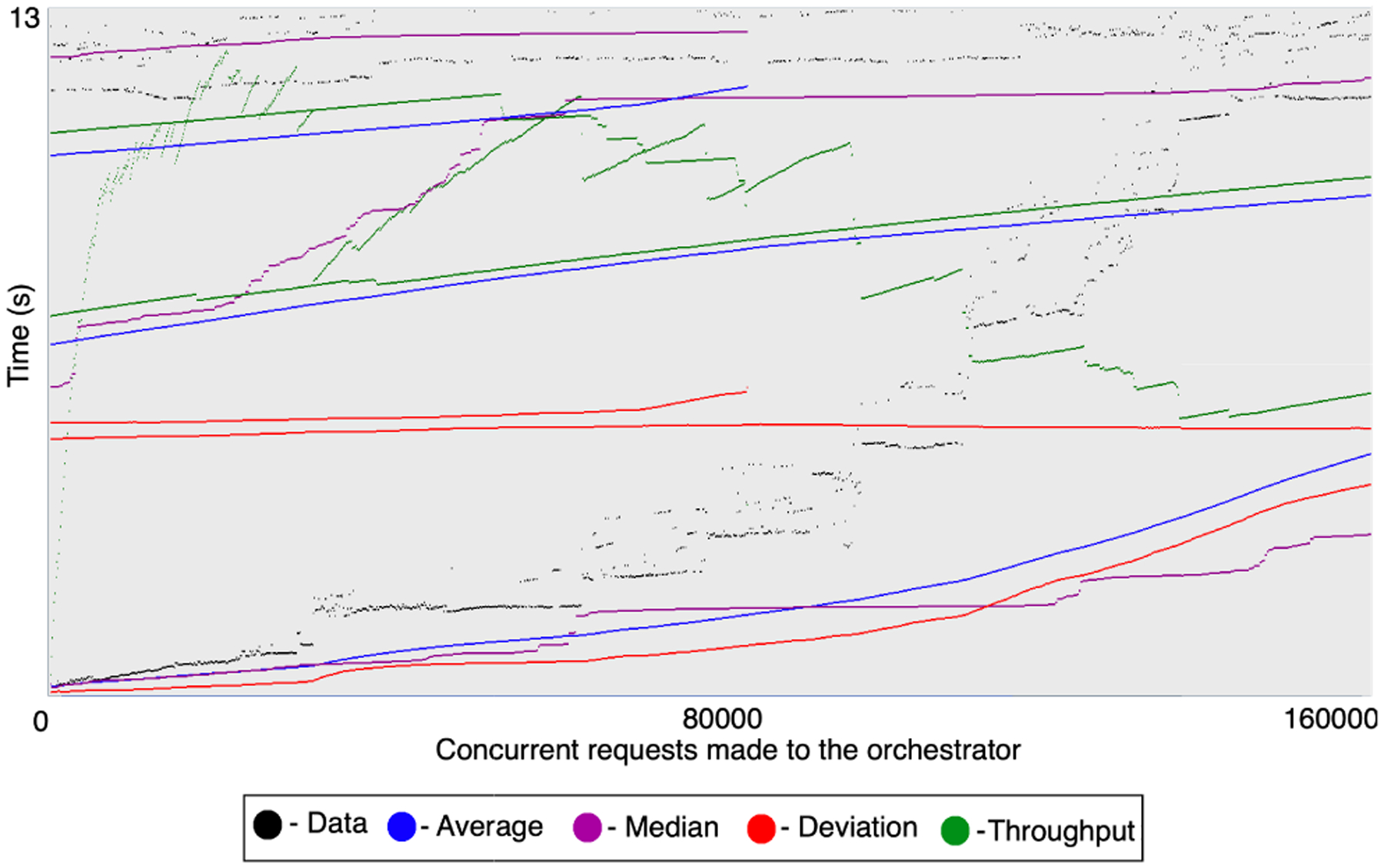
Execution of 5000 concurrent service workflows via the *NEXUS* orchestrator.

Figure 12 shows the performance with 5000 such concurrent workflows with a startup period of 10 s for all the invocations. We observe the throughput to be 3412.39 workflow invocations per minute. The orchestrator consumes 11.945 s on average, 13.015 s on median, and 6.008 s of deviation. As the requests exceeded the x-axis in the representation, Jmeter shows an overlapping plot after the 160,000 concurrent requests. We note how the orchestrator manages to scale up with the problem size. Its throughput increases with more concurrent workflow invocations without incurring data loss in the control flows. Although all the 1000 workflow executions succeeded with the 1000 concurrency, 4772 workflows out of 5000 succeeded with the concurrency of 5000 due to timeouts in a few service requests.

We note that with the startup period of 1 s, *NEXUS* handles the concurrency of 1000 workflows well. It starts to incur delayed control flow responses when we increase the workflow execution concurrency to 5000, even with the increased startup period of 10 s. Here, 5000 workflows are scheduled simultaneously within 10 s for the orchestrator to manage the control flows. The delayed response at 5000 concurrency leads to the failure of a few workflow executions as a few of the services start to time out while waiting for the orchestrator. We notice 228 failed workflow executions out of 5000, with a 95.44% success rate compared to the 100% success rate with 1000 concurrent workflows. This observation highlights that while the orchestrator’s performance was sufficient to handle the concurrency of 1000 workflows elegantly, such a stand-alone orchestrator deployment is not adequate to manage 5000 workflows simultaneously. We note that increasing the startup time further, thus reducing the effective concurrency, will allow the orchestrator to manage even more workflows at once without failures.

We observed no memory or processing overhead from the server that hosts the orchestrator in both cases since the control flow and the events are lightweight. The orchestrator did not encounter errors or failures for the concurrency of 1000 and 5000 workflows. Our evaluations highlight how researchers can model complex distributed closed-loop workflows efficiently with *NEXUS* without data loss or a loss in workflow performance, smoothly scaling with the problem size and concurrency.

## V. RELATED WORK

In this section, we evaluate the state-of-the-art on service interoperability and workflows.

### A. INTEROPERABILITY

Global Alliance For Genomics & Health (GA4GH) [22] and the Open Bioinformatics Foundation [23] are steering the research and interoperability effort on workflow definitions for biomedical informatics. Popular workflow languages such as CWL [2], WDL [24], and NextFlow [25] help develop scientific applications in a modular fashion from interoperable services. Scientific research use cases include developing reproducible analysis workflows for genomics [26] and creating bioinformatics workflows [27]. While workflow languages and frameworks are widely used in science, their functionality is limited to support the DAG workflows typically. They do not natively support closed-loops. *NEXUS* is a workflow orchestrator that facilitates DHG workflows with its compact model to compose workflows using workflow languages as well as from stand-alone services.

### B. OPEN-SOURCE WORKFLOW FRAMEWORKS

Among the CWL and WDL frameworks, Toil [12], CWL-Airflow [13], and AWE [28] offer complete support for CWL, potential to create workflows from containerized services, scalable execution locally, as well as across the popular cloud platforms, with auto-scaling support. Toil and CWL-Airflow are also actively developed in Python. Toil has full support for CWL as well as experimental support for WDL. Cromwell has full support for WDL, and its recent versions support CWL as well. Although Toil does not have a drag-and-drop front-end, Rabix [29] consists of a visual editor for CWL workflows, supporting drag-and-drop of service components to compose workflows. Rabix could be used in conjunction with other more complete CWL frameworks such as Toil to get the best of both worlds: i) using Rabix as a front-end to create simple workflows from services and then create complex workflows from the simple workflows, ii) save the services as CWL, and finally iii) use a CWL framework such as Toil to deploy and execute the workflows on-premise as well as on cloud environments. We also evaluated other workflow frameworks such as Apache Airavata [30], Apache Taverna [31], and Spotify Luigi [32]. However, they fall short due to their vendor-specific implementation with restricted support to standard workflow languages and interoperability across existing biomedical informatics workflow definitions.

### C. BUSINESS PROCESS WORKFLOWS

Business Process Execution Language (BPEL) [33] is a more flexible alternative to define workflows, focusing on enterprise business processes rather than eScience workflows. BPEL is based on classic light-weight web services, typically developed with the SOAP messaging protocol [34]. The BPEL specification is written in an XML-based Web Service Description Language (WSDL) [35]. However, these technologies are not commonly used in the biomedical informatics domain. Furthermore, these classic web services are executed in web service engines such as Apache Axis2 [36] and Apache CXF [37] instead of running them with Docker containers or locally as microservices. The dependence on XML, SOAP, and WSDL makes adapting BPEL to control systems modeling difficult and inefficient. Research has studied the DHG-based representation for workflows [38], scheduling [6], and resource allocation [39]. However, such representations limit their focus to business processes rather than scientific workflows [40]. Due to this state of affairs, the applicability of BPEL and research work that focuses on business processes to control systems is largely limited. Furthermore, unlike *NEXUS*, these approaches do not consider such representation to facilitate composing and managing diverse workflows from existing services and workflows in a flexible and distributed manner.

### D. SDN FOR SERVICE COMPOSITION WORKFLOWS

Previous works have elaborated how SDN can help achieve context-aware service compositions [41]. While those research works ensure Quality of Service (QoS) in workflows by leveraging SDN, they also limit their focus to DAG workflows rather than providing a flexible solution covering workflows with dicycles. Furthermore, they are approaches that use an SDN controller to ensure QoS, rather than using a software-defined system in composing the workflows dynamically. There are also event-driven workflow frameworks that allow the dynamic creation of workflows based on events [42]. However, these event-driven and SDN research works focus on DAG workflows rather than natively supporting flexible DHG workflows.

### E. WORKFLOW SIMULATIONS

Researchers have developed simulations for feedback systems and closed-loop controls for various research domains [43]. Similarly, flexible event-driven simulators [44] model executions driven by events. However, these simulators do not execute an actual service workflow. Unlike simulators and emulators that merely simulate or emulate an execution, a workflow framework indeed executes the workflow. As a workflow orchestrator, *NEXUS* executes the workflows rather than simply simulating them.

## VI. CONCLUSION

This paper presents *NEXUS*, a framework that orchestrates complex workflows with loops, composed of services and simple workflows. SDN inspired the Software-Defined Workflows of *NEXUS*. The *NEXUS* orchestrator centrally coordinates the service nodes and manages workflows dynamically. We deployed *NEXUS* to run closed-loop neuromodulation control systems natively. Our evaluations highlight the efficiency of designing and running complex workflows with *NEXUS*, from services implemented in various programming languages and workflows of standard languages such as CWL and WDL. We also illustrate the potential to design control systems as decoupled workflows spanning multiple infrastructures and platforms with *NEXUS*. We thus highlighted the scalability of *NEXUS* in the presence of several concurrent workflows.

Although *NEXUS* can support inter-organization workflows, leveraging the orchestrator to build and manage workflows for several organizations in production requires two additional considerations. First, we must incorporate security measures into the orchestrator to ensure the workflow definitions are free from malicious entities attempting to alter an executing workflow. We also must secure the orchestrator against denial of service attacks. Second, privacy measures must be in place if the services are shared across organizations to compose a workflow with *NEXUS*. Such extra steps enable deploying *NEXUS* in a multitenant edge or a hybrid cloud environment for multiple organizations. As future work, we propose leveraging *NEXUS* to execute workflows composed of services maintained by various organizations.

## Figures and Tables

**TABLE 1.** *NEXUS* notation.

Notation	Description
O	The Orchestrator
W	A workflow
Φ ∈ {A, B, C, …}	A service
*ϕ* ∈ {a, b, c, … }	A service instance
$\forall n\in {\mathbb{Z}}^{+}$, *ϕ*^*n*^	*n*^*th*^ iteration of *ϕ*
*ϕ*_*n*_	*n*^*th*^ instance of Φ for *ϕ*^*n*^
A→B	A unidirectional edge
A↔B	A loop, i.e., a bidirectional edge
$N\in {\mathbb{Z}}^{+}$	Iteration when the exit condition is met
**w** ^n^	*n*^*th*^ iteration of W
